# Scopolamine-Induced Amnesia in Zebrafish: Behavioral Characterization and Pharmacological Reversal

**DOI:** 10.3390/ani15172624

**Published:** 2025-09-08

**Authors:** Myrna Déciga-Campos, Janet Siles-Guevara, Susana Alejandra Gil-López, Jennifer Pineda-Oliveros, Rolffy Rubén Ortíz-Andrade

**Affiliations:** 1 Sección de Estudios de Posgrado e Investigación, Escuela Superior de Medicina, Instituto Politécnico Nacional, Plan de San Luis y Díaz Mirón s/n Col., Casco de Santo Tomás, Ciudad de México 11340, Mexico; 2 Facultad de Química, Universidad Autónoma de Yucatán, Colonia Inalámbrica, Mérida Yucatán 97069, Mexico

**Keywords:** scopolamine, donepezil, haloperidol, cognition, anxiety, Alzheimer’s, *Danio rerio*, zebrafish

## Abstract

The main objective of this study was to assess whether donepezil or haloperidol, after a 10-day training period, could inhibit inhibitory avoidance and alter learned behavior. To assess memory impairment, zebrafish were trained in a light/dark tank over a 20-day period: 10 days without mechanical stimulation and 10 days with mechanical stimulation. Three behavioral parameters were recorded, i.e., latency to enter the dark compartment, time spent in the dark zone, and the number of entries into the dark area. Following the training phase, scopolamine was administered to induce amnesia. Donepezil and haloperidol were used as reference compounds for comparison. Subsequently, zebrafish were placed in a cylindrical tank, and spontaneous exploratory behavior was evaluated by measuring the amount of time spent in the upper region of the tank, an indicator of anxiety-related behavior. The behavioral data from the training phase were used to establish a baseline profile of zebrafish performance in the light/dark tank. Scopolamine administration significantly altered all of the measured behavioral parameters, including exploratory behavior in the cylindrical tank. Notably, donepezil or haloperidol prevented the memory-impairing effects of scopolamine, supporting the use of the light/dark tank paradigm to evaluate the efficacy of pharmacological agents in reversing scopolamine-induced amnesia in zebrafish.

## Simple Summary

The use of zebrafish (*Danio rerio*) has become increasingly common in the experimental pharmacology field due to their viability as an *in vivo* model. Behavioral assessment in this species offers a practical and cost-effective approach for experimental design. In this study, our objective was to assess whether donepezil or haloperidol, after a 10-day training period, could inhibit inhibitory avoidance and alter learned behavior. For this purpose, we characterized the baseline behavior of zebrafish and their behavioral response in the presence of an amnestic agent, such as scopolamine, in a light/dark tank test. The results show that a standardized behavioral model in this species allows for the efficient evaluation of a larger number of pharmacological compounds. It facilitates the identification of specific therapeutic targets that can later be validated in higher-order animals, such as rodents. Preclinical pharmacological screening in non-mammalian vertebrates contributes to the reduction in the use of mammals in research, in alignment with the ethical principles of replacement, reduction, and refinement (3Rs), while enabling the identification of molecular targets with specific activity. Behavioral quantification represents a straightforward observational method that can be readily implemented in academic settings lacking specialized infrastructure, thereby broadening the opportunities for contributions to drug development from a broader range of scientific and educational institutions.

## 1. Introduction

Cognitive disorders, particularly those associated with neurodegenerative diseases such as Alzheimer’s and Parkinson’s disease, are characterized by progressive impairments in memory, learning, and executive functions. These deficits have been strongly linked to cholinergic dysfunction, especially within the hippocampus and related brain regions. While Alzheimer’s disease is primarily recognized for its cognitive decline, it is also accompanied by neuropsychiatric symptoms, including psychological distress, anxiety, and depression [1].

Scopolamine, a non-selective muscarinic acetylcholine receptor antagonist, is widely employed in preclinical research to induce transient amnesia and to model cognitive impairment associated with cholinergic deficits [2]. By disrupting cholinergic neurotransmission, scopolamine provides a reliable experimental framework for evaluating potential cognitive enhancers or neuroprotective agents [2].

Traditionally, rodent models have been used in memory research; however, zebrafish (*Danio rerio*) have recently garnered increasing recognition as a powerful and complementary model in neuroscience. Zebrafish offer practical advantages—including small size, high fecundity, rapid development, and transparent larvae—which facilitate *in vivo* pharmacological screening. Importantly, *D. rerio* shares extensive genomic, and neurochemical homology with mammals, including approximately 70% similarity with the human genome and expresses orthologs of genes implicated in human neurological disorders [3,4].

Learning, defined as the acquisition of new information, and memory, defined as the consolidation, storage, and retrieval of such information, are highly interdependent processes that are often difficult to study in isolation [5]. Zebrafish display complex cognitive functions—including preference, learning, memory, and habituation—which can be evaluated using paradigms such as the novel tank test, T-maze, Y-maze, and the light/dark preference test [6,7]. Although maze-based paradigms enable the study of multiple behavioral domains (e.g., anxiety, learning, memory), methodological differences in protocol design can yield variable interpretations [7]. Among the simplest forms of learning, habituation represents a reduction in response following repeated stimulation (non-associative learning), while avoidance paradigms assess associative learning. Both kinds of learning have been demonstrated in diverse organisms, from invertebrates to rodents [8,9,10,11].

The light/dark box is widely used for the evaluation of anxiety and cognition in rodents [11,12], but little is known about the light/dark preference in zebrafish. Its application for evaluating learning with specific conditioning has been limited [11,12]. In this study, we adapted the light/dark paradigm to assess cognition in zebrafish, highlighting its potential as a tool for evaluating novel strategies aimed at cognitive recovery. This model allows for the quantification of memory performance through latency, number of entries, and time allocation across compartments.

Th zebrafish model provides an effective platform to investigate the amnesic effects of scopolamine and to explore mechanisms relevant to human neurodegeneration due to its conserved cholinergic system and well-characterized behavioral repertoire [13]. Furthermore, it facilitates early-stage pharmacological screening to identify neuroactive compounds with potential therapeutic applications. The growing prevalence of neurodegenerative disorders, such as Alzheimer’s and Parkinson’s disease, has intensified the search for experimental models that allow a better understanding of their underlying mechanisms and the identification of effective therapeutic strategies.

The zebrafish model offers multiple advantages, such as rapid development, transparency during early life stages, ease of maintenance and reproduction, and the capacity for high-throughput behavioral and pharmacological screening [14]. The zebrafish’s well-characterized central nervous system and its sensitivity to pharmacological agents make it particularly valuable for modeling cognitive dysfunctions and evaluating potential nootropic or amnesic compounds. Recent studies have validated its use in exploring the molecular and cellular bases of central nervous system disorders, including Alzheimer’s disease, Parkinson’s disease, Huntington’s disease, and amyotrophic lateral sclerosis [15,16]. Among the compounds of interest in memory research, scopolamine—a non-selective muscarinic acetylcholine receptor antagonist—has been widely used to induce transient cognitive impairments in various animal models, including zebrafish, simulating aspects of cholinergic dysfunction observed in human neurodegeneration.

The main objective of this study was to determine the amnesic effect of scopolamine in zebrafish by employing a behavioral training paradigm in a light/dark tank. To demonstrate the cognition effect, we used donepezil and haloperidol as reference drugs. This approach allows for the assessment of learning and memory processes through quantifiable behavioral metrics, providing insights into the neuropharmacological basis of memory deficits and offering a platform for future therapeutic screening.

## 2. Materials and Methods

### 2.1. Experimental Animals

Adult zebrafish (*Danio rerio*) (body weight: 500–900 mg; length: 2.0 ± 0.5 cm; six months of age) were obtained from a local commercial supplier and inspected by an expert aquaculturist prior to inclusion in this study. The fish were maintained at a density of one fish per liter in aerated housing tanks (90 × 40 × 37 cm) equipped with activated carbon filters and air pumps. The tanks were kept under semi-natural conditions with a room temperature range of 33–35 °C and a 12:12 h light/dark cycle (lights on from 6:00 a.m. to 6:00 p.m.) for a minimum of six weeks before the onset of experiments.

Water quality parameters were strictly controlled in accordance with the zebrafish husbandry guidelines: water temperature: 27.5 ± 0.5 °C; pH: 7.0 ± 0.5; dissolved oxygen: 6 ± 0.1 mg/L; total ammonia: <0.01 mg/L; total hardness: 6 mg/L; and alkalinity: 22 mg/L CaCO_3_ [17]. Fish were fed ad libitum with commercial tropical fish flakes (Biomaa S.A. de C.V., Jilotzingo Estado de Mexico, Mexico) and *Artemia salina* nauplii cultured from freeze-dried eggs, following previously established methods [18].

All experimental procedures complied with the Mexican Official Norm for Animal Care and Handling (NOM-062-ZOO-1999), the ARRIVE guidelines, the UK Animals (Scientific Procedures) Act 1986, the EU Directive 2010/63/EU, and the U.S. National Research Council’s Guide for the Care and Use of Laboratory Animals. The study protocol was approved by our institution (Approval No. SIP-2023-1277). Fish were randomly assigned to experimental groups (*n* = 8 per group). All efforts were made to minimize animal distress and reduce the number of animals used. After experimentation, animals were euthanized by immersion in cold water (2–4 °C). All procedures, including training and treatment, were performed individually.

### 2.2. Reagents

Donepezil [2-[(1-benzylpiperidin-4-yl)methyl]-5,6-dimethoxy-2,3-dihydroinden-1-one], haloperidol [4-[4-(4-chlorophenyl)-4-hydroxypiperidin-1-yl]-1-(4-fluorophenyl)butan-1-one], scopolamine [(1R,2R,4S,5S)-9-methyl-3-oxa-9-azatricyclo [3.3.1.02,4]nonan-7-yl] (2S)-3-hydroxy-2-phenylpropanoate] and fluoxetine [N-methyl-3-phenyl-3-[4-(trifluoromethyl)phenoxy]propan-1-amine] were purchased from Sigma-Aldrich (St. Louis, MO, USA). All compounds were dissolved in tank water and administered in one-liter tanks containing four fish each.

### 2.3. Scopolamine-Induced Memory Impairment

#### 2.3.1. Dark Zone Training

Zebrafish were conditioned in a light/dark preference tank (20 × 25 × 13 cm; water depth: 3 cm) separated into two compartments by a movable central acrylic gate. One compartment was translucent, while the other was lined with black, non-reflective material.

**Figure 1:**
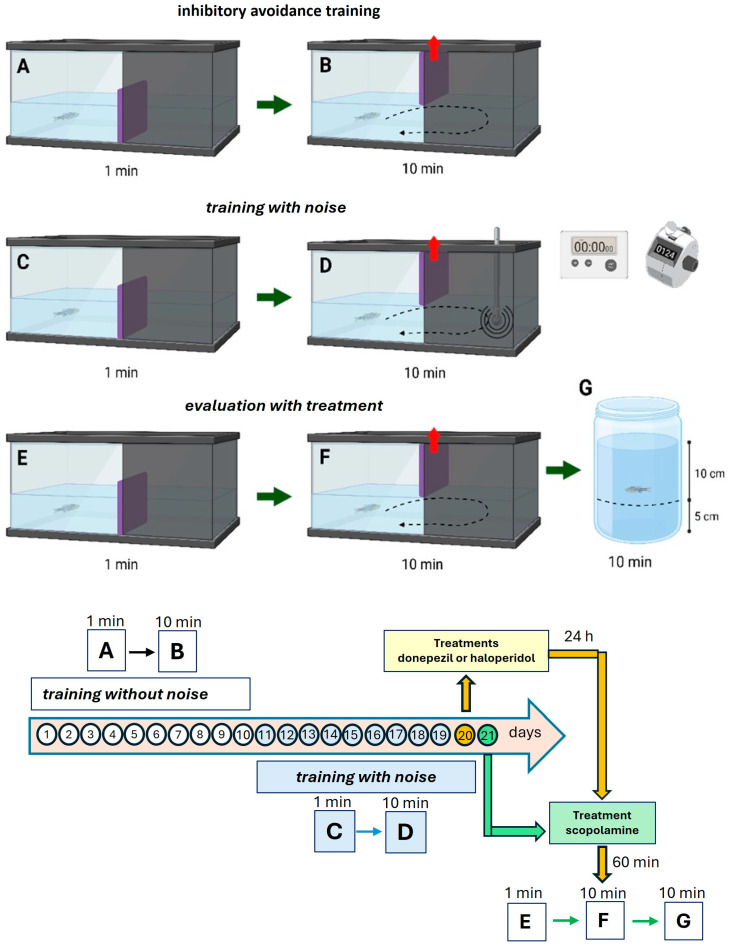
Experimental setup, including a light/dark tank and mechanical noise, used to measure cognition in zebrafish. Behavior was evaluated for ten days in the absence of mechanical noise (**A**,**B**). Afterward, behavior was evaluated for ten days, during which mechanical noise was produced each time the zebrafish entered the dark zone (**C**,**D**). On day 21, the zebrafish were exposed to scopolamine one hour before their behavior was evaluated in the absence of mechanical noise (**E**,**F**); afterward, the zebrafish were placed in a cylinder tank, i.e., a new environment (**G**). Each fish was assessed individually for 11 min every day; the fish were kept in the light zone for one minute before the door to the dark zone was opened. The evaluation time in the new environment was also ten minutes. The zebrafish trained with noise were immersed in donepezil (2 μM) or haloperidol (1 μM) for 24 h before they were immersed in scopolamine (200 μM). Other zebrafish groups without training with noise were immersed in donepezil (2 μM) or haloperidol (1 μM), scopolamine, or fluoxetine for 24 h before introducing them into a novel environment.

In the first training phase, each fish was individually placed in the light compartment for 1 min with the gate closed (Figure 1A). The gate was then opened, allowing free movement into the dark compartment for 10 min. During this period, three behavioral parameters were recorded, i.e., (1) latency to enter the dark zone, (2) number of entries into the dark zone, and (3) time spent in the dark zone. This training was conducted daily for ten consecutive days (Figure 1B).

#### 2.3.2. Inhibitory Avoidance Task

A homogenizer was used to deliver aversive mechanical noise in the dark zone for fear conditioning, following Rajesh et al.’s method, [19]. The stirrer was submerged 1 cm into the water within the dark compartment (operated at a speed of 8000 rpm, producing a noise level of 70 dB). This noise was activated each time the fish entered the dark area of the tank and was turned off immediately upon leaving it.

On day 11, the same procedure as in the first phase was followed (Figure 1C). However, the homogenizer was activated when the fish entered the dark compartment, inducing aversive conditioning and prompting the fish to move toward the light zone. The noise ceased when the fish exited the dark zone. This aversive training continued for ten days (Figure 1D). All sessions were video-recorded, and fish behavior was assessed independently by two observers to minimize bias.

#### 2.3.3. Scopolamine-Induced Cognitive Deficit

On day 21, following inhibitory avoidance training, zebrafish were immersed in a 200 μM scopolamine solution for one hour [14,15]. Behavioral assessments were performed after training in the light/dark tank (Figure 1E,F).

#### 2.3.4. Behavioral Assessment in a Novel Environment

To evaluate exploratory behavior, trained fish were transferred to a cylindrical tank (23 cm height × 9.5 cm diameter) filled with water to a depth of 15 cm. The tank was conceptually divided into two zones: an upper zone (10 cm) and a lower zone (5 cm). Time spent in the upper zone was recorded for 2 to 10 min post-transfer (Figure 1D). Data were analyzed as changes over time.

#### 2.3.5. Experimental Design

In the first experiment, four groups were evaluated based on exposure to mechanical noise. On day 21, two groups (one with and one without noise exposure) were treated with scopolamine (200 μM, one hour). The remaining two groups received naïve treatment (tank water) and were similarly categorized based on prior noise exposure. Behavioral outcomes were assessed as previously described (Figure 1).

In the second experiment, all fish underwent 20 days of light/dark training with mechanical noise exposure. On day 20, fish were divided into groups (*n* = 8/group). One group was treated with donepezil (2 μM, 24 h) and another with haloperidol (1 μM, 24 h), and there was also a naïve group. On day 21, behavior was evaluated in the light/dark tank.

In the second part of the experiment, fish from each pharmacological group were subsequently exposed to scopolamine (200 μM, one hour) on day 21 prior to behavioral testing. Data were compared with the naïve-treated control group from the first experiment. Notably, higher doses (4 μM donepezil and 2 μM haloperidol) were found to be toxic.

In another set of experiments, groups of fish that were not subjected to noise training or the light/dark tank were exposed to donepezil (2 μM), haloperidol (1 μM), scopolamine (200 μM), or fluoxetine (1 μM) for 24 h prior to the assessment with a novel environment.

## 3. Results

### 3.1. Baseline Behavior of D. rerio in the Light/Dark Tank

This study reports the amnesic effects of scopolamine on *Danio rerio* using a light/dark tank avoidance paradigm and a novel environment (cylindrical tank) test. Initially, naïve fish were placed in the light zone of a two-chamber tank, and after one minute, a central gate was opened to allow free access to the dark zone. Behavioral parameters recorded over 10 min included the following: (1) latency to enter the dark zone, (2) number of entries into the dark zone, and (3) time spent in the dark zone. Subsequently, the fish were transferred to a cylindrical tank to assess exploratory behavior, with the duration of time spent in the upper half of the tank recorded for 10 min.

**Figure 2:**
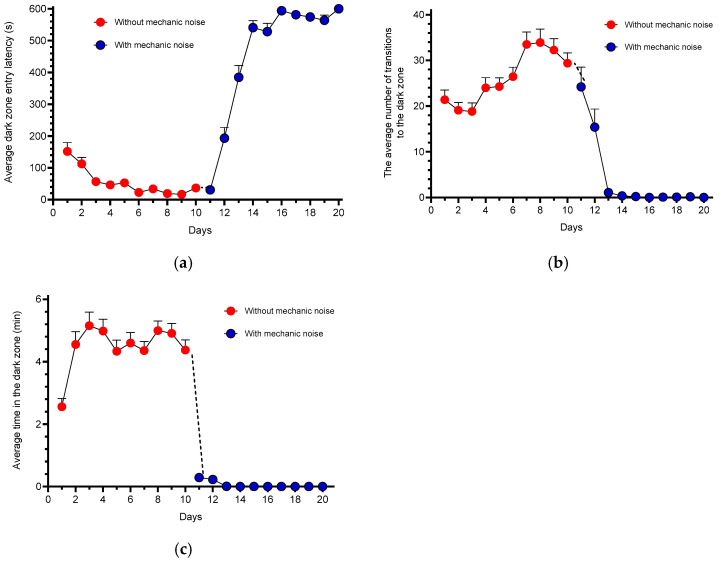
*D. rerio* behavior in a light/dark tank test. The time course of the average latency to enter the dark zone (**a**), the average number of entries into the dark zone (**b**), and the average time spent in the dark zone (**c**). Training was performed without mechanical noise stimulation for ten days and with mechanical noise stimulation for ten days. Each data point represents the average of 80 naïve fish ± SEM.

Figure 2 shows behavioral changes over a 20-day avoidance protocol, comprising 10 days without mechanical stimulation and 10 days with exposure to mechanical noise. During the first two days, the average latency to enter the dark zone was 180 s, which progressively declined to approximately 30 s by days 6–9. Following the introduction of mechanical noise (day 11), latency values increased sharply, reaching a maximum of 600 s on day 16 and then plateauing up to through day 20 (Figure 2a). These results indicate that the fish initially perceived the dark zone as a safe area. Rapid movements were observed in the light zone during the early training days, which subsided by day 8, coinciding with increased resting behavior in the dark compartment. However, upon exposure to mechanical noise, fish avoided the dark zone, indicating that the perceived safety was reversed.

The number of entries into the dark zone gradually increased between days 4 and 8, with a slight decline observed on days 9–10. Once mechanical noise was introduced, entries significantly decreased and remained low through day 14 (Figure 2b). The time spent in the dark zone increased on day 2 and remained consistent during the noise-free period (Figure 2c). However, a sharp reduction occurred on day 11 with noise exposure, reaching nearly zero by day 13. These findings demonstrate that zone preference in zebrafish is highly modifiable and sensitive to aversive conditioning, thus validating the utility of this avoidance paradigm for cognitive assessments. Behavioral data represent pooled results from 80 zebrafish across experiments 1 and 2.

### 3.2. Scopolamine-Induced Cognitive Impairment in D. rerio

**Figure 3:**
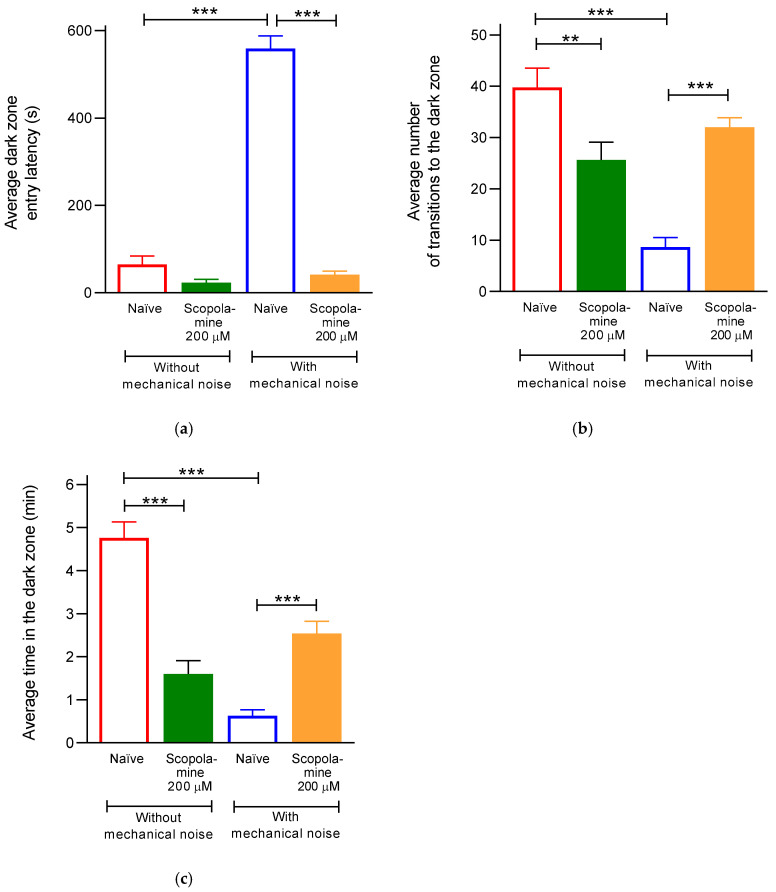
Effect of scopolamine in *D. rerio* exposed to mechanical noise. Behavior was evaluated for ten minutes after the fish were immersed in scopolamine (SCO; 200 μM/one hour) solution. The average latency to enter the dark zone (**a**), the average number of entries into the dark zone (**b**), and the average time spent in the dark zone (**c**) were quantified. Each data point represents the average of 8 fish ± SEM. One-way ANOVA (** *p* < 0.001; *** *p* < 0.0001) revealed significant differences in naïve and SCO-treated fish exposed to and not exposed to noise.

Figure 3 presents data from four experimental groups (*n* = 8 each). Two groups underwent training without mechanical noise, with one group receiving scopolamine (200 μM, 1 h) prior to behavioral testing. The remaining two groups were exposed to mechanical noise from days 11 to 20; one of these was treated with scopolamine prior to testing. In the naïve group, mechanical noise exposure increased the latency to enter the dark zone sevenfold compared to non-exposed controls. In contrast, among fish not exposed to mechanical noise, there were no significant differences in latency between naïve- and scopolamine-treated groups. However, in noise-exposed fish, scopolamine treatment significantly reduced latency compared to the noise-exposed naïve group, suggesting that scopolamine impaired the memory of the dark zone being aversive (F_(3, 32)_ = 159.2; *p* < 0.0001) (Figure 3a).

Regarding zone entries, non-noise-exposed naïve fish made an average of 32 entries into the dark zone, which was reduced by 56% in the noise-exposed naïve group. Scopolamine treatment also reduced entries in non-noise-exposed fish by 38% compared to naïve controls. Interestingly, scopolamine-treated fish exposed to noise showed a significant increase in entries (27 ± 3.4) compared to the corresponding naïve group (F_(3, 32)_ = 21.9; *p* < 0.0001) (Figure 3b), indicating amnesic effects and suggesting that the fish no longer remembered the dark zone as aversive.

Time spent in the dark zone was reduced by 96% in the noise-exposed naïve group relative to the non-exposed group. Scopolamine-treated fish not exposed to noise also showed a reduction compared to naïve controls. In contrast, noise-exposed fish treated with scopolamine spent significantly more time in the dark zone compared to noise-exposed naïve-treated fish (F_(3, 32)_ = 37.5; *p* < 0.0001) (Figure 3c), confirming the disruption of avoidance learning.

### 3.3. Donepezil Prevents Scopolamine-Induced Cognitive Impairment

**Figure 4:**
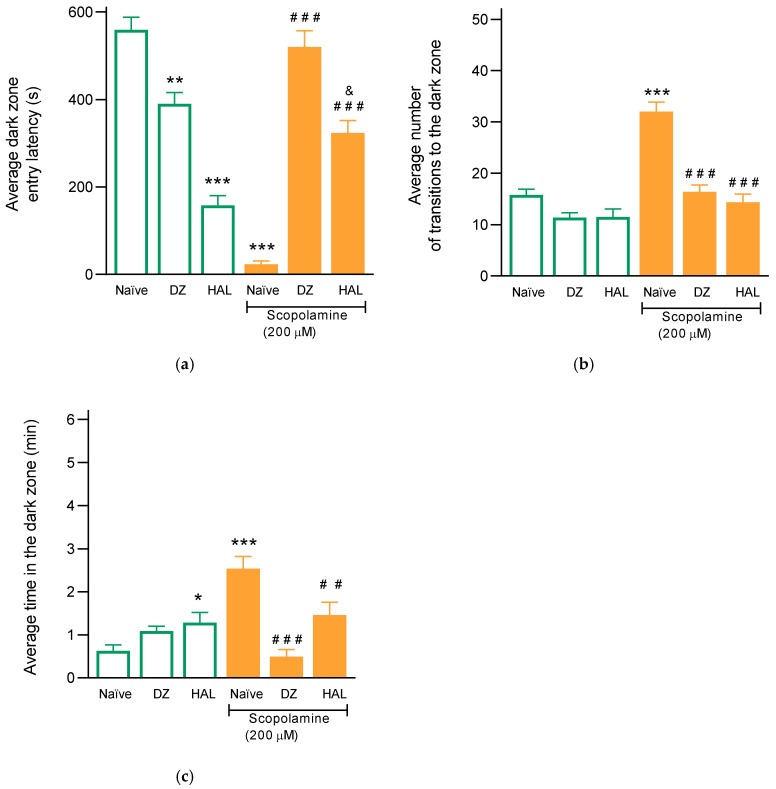
Effects of donepezil (DZ) or haloperidol (HAL) on the amnesic effect of scopolamine in *D. rerio*. Behavior presented in the light/dark fish tank: the average latency to enter the dark zone (**a**), the average number of entries into the dark zone (**b**), and the average time spent in the dark zone (**c**). Fish habituated to mechanical noise were immersed in donepezil (2 μM) or; haloperidol (1 μM) for 24 h prior to immersion in the scopolamine solution (200 μM, 1 h). Each data point represents the average of 8 fish ± SEM. (* *p* < 0.01, ** *p* < 0.001, *** *p* < 0.0001) vs. effect induced by naïve without scopolamine group; (^##^ *p* < 0.001, ^###^ *p* < 0.0001) vs. effect induced by naïve with scopolamine group; (^&^ *p* < 0.01) vs. the haloperidol without scopolamine group, two-way ANOVA followed by Tukey’s test.

Donepezil, a selective, reversible acetylcholinesterase inhibitor commonly used in the treatment of Alzheimer’s disease, was employed as a positive control. Pretreatment with donepezil (1 μM, 24 h) reduced the latency to enter the dark zone by 31% compared to noise-exposed naïve fish. In contrast, scopolamine alone reduced latency by 95%. Notably, pretreatment with donepezil significantly attenuated scopolamine-induced cognitive deficits, increasing latency by 91% compared to the scopolamine-only group (F_(5, 48)_ = 60.65; *p* < 0.0001), and restoring latency values to those observed in the naïve group (Figure 4a). Scopolamine doubled the number of entries (32 ± 1.8) compared to the naïve group (15.7 ± 1.4). Donepezil alone had no effect on this parameter. However, donepezil pretreatment prevented the scopolamine-induced increase in entries, reducing them to 11.3 ± 0.9 (F_(5, 48)_ = 26.26; *p* < 0.0001) (Figure 4b).

Scopolamine increased the time spent in the dark zone to 152.2 ± 17.2 s, while donepezil alone produced no significant change. Donepezil pretreatment before scopolamine administration significantly decreased the time spent in the dark zone to 29.5 ± 9.8 s (F_(5, 48)_ = 14.07; *p* < 0.0001) (Figure 4c). These results demonstrate that donepezil effectively reverses scopolamine-induced cognitive impairment in zebrafish.

### 3.4. Haloperidol Modulation of Scopolamine-Induced Amnesia

Haloperidol, a dopamine D2 and sigma-1 receptor antagonist, prevented the effects on scopolamine-induced behavior. In fish trained with noise and without scopolamine, haloperidol significantly decreased the latency to enter the dark zone by 77.7% (F_(5, 48)_ = 60.65; *p* < 0.0001), suggesting a potential amnesic-like effect. Haloperidol also increased the time spent in the dark zone (95.2 ± 8.4 s vs. 37.7 ± 8.3 s in naïve fish) (F_(5, 48)_ = 14.07; *p* < 0.0001) (Figure 4c). Interestingly, haloperidol decreased the number of zone entries to 11.3 ± 0.9, like the effect of donepezil, though this was not significantly different from the naïve group (Figure 4b).

In haloperidol + scopolamine-treated fish, cognitive performance improved. Latency to enter the dark zone increased to 323.4 ± 28.4 s (vs. 32 ± 1.8 s in the scopolamine group), and both the number of entries (95.2 ± 15.7 vs. 152.2 ± 17.2) and time spent (184.7 ± 21.8 s vs. 23.2 ± 7.5 s) decreased (Figure 4a–c). These findings suggest a potential interaction between haloperidol and scopolamine, although the precise nature of this interaction remains unclear.

### 3.5. Behavioral Assessment in a Novel Environment After Light/Dark Tank Training

**Figure 5:**
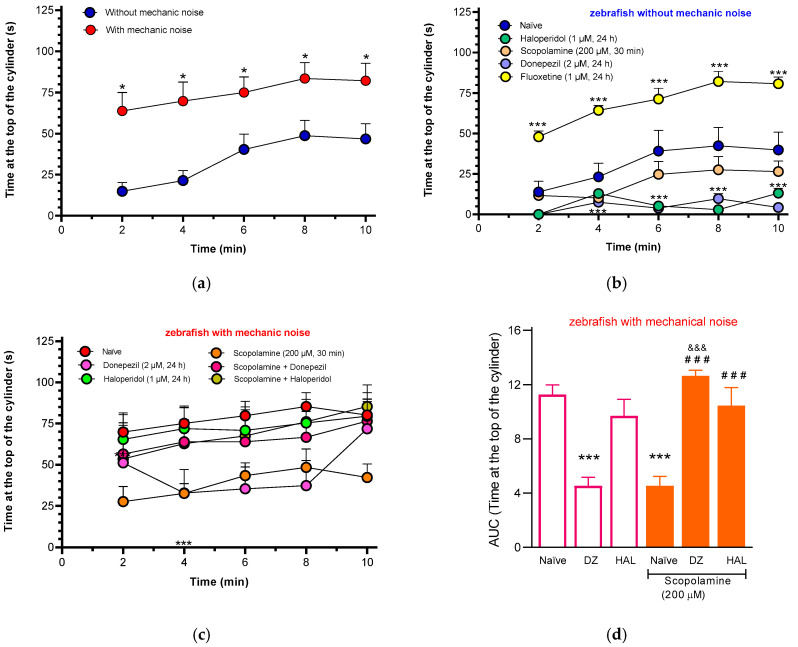
*D. rerio* behavior in an exploration cylinder. The fish were evaluated in a new environment after the light/dark tank test training. The behavior was quantified as the time spent in the upper part of the cylinder every two minutes for a period of ten minutes. Zebrafish exposed with or without mechanical noise training (**a**). Temporal course of zebrafish without mechanical noise training that were immersed in donepezil (2 μM, 24 h), haloperidol (1 μM, 24 h), scopolamine (200 μM, 1 h), or fluoxetine (1 μM, 24 h) (**b**). Temporal course of zebrafish that were immersed in donepezil (2 μM), haloperidol (1 μM) for 24 h prior to immersion in the scopolamine solution (200 μM, 1 h) following mechanical noise training (**c**). The bars represent the AUC obtained from the Temporal course of zebrafish that were immersed in donepezil (2 μM), haloperidol (1 μM) for 24 h prior to immersion in the scopolamine solution (200 μM, 1 h) following mechanical noise training (**d**). Each data point represents the average of 8 fish ± SEM. (* *p* < 0.01, *** *p* < 0.0001) vs. effect induced by naïve group; (^###^ *p* < 0.0001) vs. effect induced by scopolamine; (^&&&^ *p* < 0.0001) vs. the donepezil without scopolamine group, two-way ANOVA followed by Tukey’s test.

Light/dark tests are among the most widely used models for studying and quantifying anxiety in zebrafish. These assays are based on the species innate defensive behavior when exposed to a novel and potentially threatening environment [20]. Memory can be assessed as a decrease in response to an aversive stimulus, such as electric shocks [21], as well as by an increase or decrease in the expression of a specific behavior following the application of a positive or negative reinforcement/punishment (e.g., when a fish learns to explore a zone in a novel environment). To assess exploratory behavior and confirm the absence or presence of anxiety-like behavior, zebrafish were placed in a cylindrical tank post light/dark tank testing. Figure 5a shows that fish not exposed to mechanical noise spend more time in the lower zone (14.9 ± 5.2 s/120 s) at the start and slowly explore more at the end of the trial (46.7 ± 5.2 s/120 s). In contrast, fish exposed to mechanical sounds spend more time in the upper region from the start (63.7 ± 18.4 s/120 s) and maintain this behavior for the entire trial (82 ± 23.1 s/120 s). In this sense, fish that were subjected to noise appear to have less anxiety (AUC = 602.1 ± 27.3 au) than those that did not receive noise training (AUC = 282.5 ± 23.2 au), based on the swimming time in the upper zone.

Figure 5b shows that fish not trained with noise and exposed to haloperidol (AUC = 54.98 ± 3.9 au), donepezil (AUC = 46.39 ± 4.1 au), or scopolamine (AUC = 102.8 ± 13.1 au) spent more time swimming in the lower part of the tank compared to those in the naïve group (AUC = 282.5 ± 23.2 au). Noteworthily, although fish from all three groups swam below the standard baseline of zebrafish behavior, their patterns differed. For instance, in the scopolamine group, fish exhibited reduced locomotor activity and remained inactive primarily near the bottom. In contrast, fish exposed to donepezil, while also swimming below 5 cm in the cylinder, displayed high levels of activity. Haloperidol-treated fish demonstrated a distinct pattern characterized by frequent vertical movements. However, they predominantly remained in the lower zone, and they repeatedly explored the upper part of the tank through rapid upward and downward swimming. Donepezil and haloperidol could be related to the anxiogenic effect in zebrafish. Contrarily, fluoxetine, a drug commonly used to treat anxiety, produced a twofold increase in swimming time in the upper part of the cylinder (AUC = 563.5 ± 11.1 au) compared to the naïve group. We used this drug as a positive control to demonstrate that an anxiolytic effect is characterized by prolonged swimming in the upper zone of the cylinder.

Pretreatment with donepezil and mechanical stimulus exposure decreased the AUC by 40% in zebrafish, whereas haloperidol alone did not alter this behavior relative to the naïve group without scopolamine. However, in the combined treatment with donepezil in the presence of scopolamine, the time spent in the upper zone increased (12.6 ± 0.4 au), showing a significant difference from the scopolamine group (F_(5, 48)_ = 31.95; *p* < 0.0001). Haloperidol did not modify its effect in the presence of scopolamine, with no significant differences between the haloperidol/scopolamine and haloperidol-only groups. Nevertheless, haloperidol prevented the scopolamine-induced reduction in time spent in the upper zone (Figure 5c,d). These data suggest that noise-trained fish tend to swim at higher levels in the cylinder tank, an effect that was altered by scopolamine, which induced amnesia. The treatments (scopolamine, donepezil, and haloperidol) were unlikely to be associated with anxiety-related behavior because they did not increase the time spent by the fish in the upper zone in the absence of mechanical noise exposure. Although further studies are required to elucidate the underlying neural mechanisms, our findings support the potential utility of this protocol for investigating learning and memory processes.

## 4. Discussion

In this study, we employed the light/dark tank paradigm to investigate the cognitive effects of scopolamine in adult zebrafish (*Danio rerio*) habituated to mechanical noise. Initially, fish were trained to recognize the dark compartment as a safe zone. Following this training phase, mechanical noise was introduced to induce aversive conditioning, discouraging entry into the dark zone. Three behavioral parameters were quantified to assess cognitive performance (1) latency to enter the dark zone, (2) number of entries into the dark zone, and (3) total time spent in the dark zone. These variables have previously been validated as indicators of anxiety-like and cognitive behavior in zebrafish [22].

In this context, we used scopolamine to disrupt previously acquired behavior over a 20-day training protocol in the presence of mechanical noise. This form of learning, classified as non-associative, arises in response to repeated exposure to a single stimulus without reinforcement and has been demonstrated across multiple species, including *Aplysia*, *Tritonia*, *Drosophila*, and *rodents* [23].

Phototactic and scototactic responses in zebrafish remain controversial [24,25,26], though rodent studies have consistently shown a preference for dark environments due to their nocturnal nature [27,28]. In our study, zebrafish displayed a clear preference for the dark compartment under baseline conditions, aligning with previous observations linking this behavior to anxiety modulation [20]. However, when mechanical noise was introduced, the fish exhibited a shift in preference toward the light zone, suggesting successful aversive conditioning. Notably, this learned behavior was reversed upon immersion in scopolamine, supporting its established role as a pharmacological agent capable of inducing amnesia.

Scopolamine, a non-selective muscarinic receptor antagonist, is widely used in experimental models to induce transient cognitive impairment analogous to the deficits observed in dementia [24]. It disrupts cholinergic neurotransmission primarily through antagonism of M1 muscarinic receptors in the cortex, resulting in dysregulated acetylcholine release and neuronal loss in the hippocampus [29,30]. Additionally, NMDA receptor involvement has been implicated in scopolamine-induced hippocampal dysfunction [31].

Topical drug delivery via immersion in zebrafish is an efficient non-invasive method that facilitates compound absorption through the gills and systemic distribution across the blood–brain barrier [32]. Lipophilicity is a critical determinant of drug uptake, as evidenced by the differential bioaccumulation of haloperidol (logP = 4.3) versus scopolamine (logP = 0.98) [33].

Our findings prove for the first time that zebrafish exposed to aversive stimuli in the light/dark tank may represent a valuable approach for evaluating cognition. The behavioral alterations induced by scopolamine—characterized by reduced latency, increased number of entries, and prolonged duration in the dark zone—are consistent with cognitive impairments and support its use as an amnesic model. While the light/dark test has been extensively employed to assess anxiety-like behavior in both zebrafish [34] and rodents [35], our results suggest that modifications of this paradigm may broaden its applicability to the study of cognitive processes.

Upon mechanical noise exposure, fish exhibited reduced activity and avoided the previously preferred dark zone. The subsequent restoration of this behavior following scopolamine treatment suggests impaired cognition towards aversive conditioning. Whether this behavioral restoration is attributable to memory loss or anxiolytic effects remains under investigation. Classical anxiety indicators in zebrafish include reduced exploration of the upper tank, increased freezing, and erratic movement, which may overlap with cognitive impairment symptoms [35,36,37]. Our data from the cylinder test suggest that the effects of scopolamine could be related to a cognitive process. In fish not trained with mechanical noise, scopolamine reduced locomotor activity and produced slower swimming in the cylinder; this effect was not different from that observed in naïve fish. However, under noise conditions, the scopolamine effect was statistically different from that observed in untreated control fish.

Currently, we do not have evidence of how relevant this amnesic effect of scopolamine is when the fish are in a novel environment, because the fish have no prior learning about the cylinder. Training fish in an inhibitory avoidance setup probably changes receptor expression in certain brain regions of the fish, which results in brave behavior (fish stay in the bright area, which normally is fear-inducing). However, by adding scopolamine, these brain regions are inhibited, causing the fish to return to their previous anxious state. This observation results from both the effect of using a novel tank and the inhibitory avoidance of fish.

In normal, untrained fish, noise exposure reduced the effects of haloperidol, scopolamine, and donepezil, suggesting a possible anxiogenic influence, contrary to fluoxetine. These responses differed from those observed in fish trained with mechanical stimulation. Taken together, these findings support the interpretation that the observed effects are more closely associated with cognition than with anxiety-related behavior. However, it is necessary to establish which molecular mechanisms are involved in these behavioral responses.

To further explore the cognitive implications, we employed the novel tank test. Fish exposed to mechanical noise showed increased top-dwelling behavior, which was prevented by donepezil. Donepezil, a selective acetylcholinesterase inhibitor, has demonstrated efficacy in reversing scopolamine-induced deficits by restoring cholinergic tone [34]. Our results suggest that the donepezil pretreatment of adult fish prevents the scopolamine effect, which could be related to cognition; other published findings indicate that donepezil improves short-term memory and modulates the startle response in zebrafish larvae [38,39]. On the other hand, the donepezil pretreatment of adult fish is associated with an angiogenic effect, as evidenced by the increased bottom-dwelling behavior in the cylinder test, both in trained and untrained fish exposed to noise.

Interestingly, haloperidol when administered alone induced amnesic-like behavior similar to scopolamine. However, in combination with scopolamine, it reversed the amnesic phenotype. These conflicting results mirror those reported in rodent studies, where haloperidol has both failed to mitigate [40] and successfully protect against [41] stress-induced cognitive impairments.

Haloperidol’s broad receptor profile complicates interpretation. It modulates dopaminergic, cholinergic, serotonergic, and histaminergic pathways and has been shown to both inhibit and upregulate acetylcholinesterase activity depending on exposure duration [40,41,42,43]. Zebrafish possess orthologous dopaminergic components, including tyrosine hydroxylase, dopamine transporters, and D1–D4 receptors [44,45,46], enabling comparative neuropharmacological analyses with mammalian systems. Haloperidol’s effect on cognitive function may stem from its modulation of dopamine–acetylcholine interactions in the striatum [47,48].

The regional distribution and ratio of D2 autoreceptors versus heteroreceptors in dopaminergic circuits critically influence behavioral outcomes [49]. For instance, haloperidol may enhance memory retrieval via increased activation in the hippocampus and substantia nigra/ventral tegmental area (SN/VTA) while concurrently reducing prefrontal activity [50]. In zebrafish, further investigation is needed to delineate region-specific dopaminergic signaling and receptor expression patterns.

In this study, the mechanisms of action involved in the observed effect of haloperidol or donepezil were not analyzed; it is considered important to evaluate some translational mechanisms to explain the observed events.

In this study, we demonstrated that zebrafish can be useful for analyzing amnesic effects. We proposed a protocol to evaluate new treatments with potential utility in Alzheimer’s disease, as well as to assess pharmacological interventions for this condition. However, it is essential to acknowledge that, although it is a valuable model for studying neuropharmacological processes, certain translational limitations must be recognized. The zebrafish brain exhibits reduced anatomical complexity compared to the mammalian brain, which may limit the direct extrapolation of higher-order cognitive functions. In addition, differences in drug absorption, distribution, metabolism, and excretion between zebrafish and mammalian species may alter the pharmacokinetic and pharmacodynamic profiles of candidate compounds. These aspects highlight that findings obtained in zebrafish should be considered as preliminary evidence that requires further validation in mammalian models. However, zebrafish provide a cost-effective, ethically favorable, and high-throughput system that is particularly advantageous for the early stages of drug screening, prior to utilizing more complex organisms.

## 5. Conclusions

In this study, we showed that donepezil or haloperidol, after a 10-day training period, could inhibit inhibitory avoidance and alter learned behavior, suggesting that the light/dark tank could be useful for analyzing cognition using scopolamine-induced amnesia and for evaluating three behavioral parameters to assess pharmacological effects: the latency to enter the dark zone, time spent in the dark zone, and number of entries into the dark zone. Donepezil effectively prevented the cognitive deficits induced by scopolamine, demonstrating its potential to restore memory-related behavior in *D. rerio*.

## Data Availability

The original contributions presented in this study are included in the article. Further inquiries can be directed to the corresponding author(s).

## Abbreviations

The following abbreviations are used in this manuscript:

au	Area units
DZ	Donepezil
HAL	Haloperidol
AUC	Area under the curve
dB	Decibels
